# *Rickettsia felis*, an Emerging Flea-Borne Rickettsiosis

**DOI:** 10.1007/s40475-016-0070-6

**Published:** 2016-04-23

**Authors:** Lisa D. Brown, Kevin R. Macaluso

**Affiliations:** Department of Pathobiological Sciences, School of Veterinary Medicine, Louisiana State University, Skip Bertman Drive, SVM-3213, Baton Rouge, LA 70803 USA

**Keywords:** *Rickettsia felis*, Flea-borne spotted fever, Transmission biology, Epidemiology, Genetic diversity

## Abstract

*Rickettsia felis* is an emerging insect-borne rickettsial pathogen and the causative agent of flea-borne spotted fever. First described as a human pathogen from the USA in 1991, *R. felis* is now identified throughout the world and considered a common cause of fever in Africa. The cosmopolitan distribution of this pathogen is credited to the equally widespread occurrence of cat fleas (*Ctenocephalides felis*), the primary vector and reservoir of *R. felis*. Although *R. felis* is a relatively new member of the pathogenic *Rickettsia*, limited knowledge of basic *R. felis* biology continues to hinder research progression of this unique bacterium. This is a comprehensive review examining what is known and unknown relative to *R. felis* transmission biology, epidemiology of the disease, and genetics, with an insight into areas of needed investigation.

## Introduction

Insect-borne rickettsiae are among the most influential zoonotic pathogens in human populations throughout the world, with both historic (e.g., louse-borne epidemic typhus during Napoleon’s retreat from Moscow) [1] and current (e.g., reemergence of flea-borne endemic typhus in southern California and Texas) [2, 3] outbreaks. Recently, a third insect-borne rickettsial pathogen, *Rickettsia felis*, has progressed from a sporadic disease in the USA to a common cause of fever in Africa [4]. First described in 1990 from colonized cat fleas (*Ctenocephalides felis*) [5], this intracellular Gram-negative bacterium was associated with human disease by 1991 [6]. Many years passed before the species itself was formally validated by molecular criteria in 2001, and isolation of the reference strain (Marseille-URRWXCal2) from cat fleas was completed shortly thereafter in 2002 [7, 8]. The definitive description of *R. felis* as the causative agent of flea-borne spotted fever has dramatically increased the appearance of this pathogen in the literature, with roughly 315 peer-reviewed articles currently and more than 90 % of which were published after 2002. The ease of molecular tools, specifically polymerase chain reaction (PCR), to detect pathogens from around the globe has confirmed *R. felis* infections from every continent except Antarctica [4, 6, 9]. Within the last decade, several advances have been made towards the understanding of basic *R. felis* biology (e.g., genomics and pathogenicity), yet some deficiencies (e.g., transmission mechanisms, epidemiology, and species diversity) remain and continue to hinder investigative advances for this universal emerging pathogen.

### Transmission Biology of *R. felis*

Following the initial detection of *R. felis* from an isolated cat flea colony, several other commercial and institutional organizations confirmed the presence of *R. felis* in additional laboratory-reared cat flea colonies (reviewed in [10]). Sustained *R. felis* infections within cat flea populations were first postulated to occur through stable vertical transmission based on the detection of rickettsiae in flea reproductive tissues [11]. Later reports using PCR analyses confirmed vertical transmission of *R. felis* in colonized cat fleas in both freshly deposited flea eggs (transovarial transmission) and newly emerged, unfed adult fleas (transstadial transmission) [11, 12]. Subsequently, the cat flea was considered not only the primary vector of *R. felis* but also the reservoir host due to the maintenance of infection solely within the vector population [12]. Although vertical transmission has been demonstrated, prevalence of *R. felis* among cat flea colonies exhibits tremendous variability. For example, prevalence of *R. felis*-infection in adult cat fleas from a single colony ranged from 35 to 96 % over the course of 1 year [13], while comparison of F_1_ infection rates from distinct *R. felis*-infected cat flea colonies may range from 0 to 100 % based on unknown mechanisms [10]. An inverse correlation was observed between colony *R. felis*-infection prevalence and *R. felis*-infection load in individual cat-fed fleas, suggesting that vertical transmission of *R. felis* is a maintenance strategy for persistence within cat flea populations [13]; however, vertical transmission efficiency of *R. felis* in cat fleas fed on bovine blood, as opposed to cat-fed colonies, was shown to severely diminish after 12 consecutive generations [14]. The inefficient transfer of *R. felis* from adult to progeny fleas was potentially linked to the vertebrate blood source, but cat fleas lack true host specificity and *R. felis*-infected arthropods have been recovered from numerous vertebrate species (e.g., cats, dogs, rodents, opossums, hedgehogs, horses, sheep, goats, gerbils, and monkeys) [4, 10, 15]. Given that vertical transmission of *R. felis* is not 100 % efficient, it is probable that horizontal amplification is required for maintenance of this pathogen within vector populations.

Further studies with cat flea colonies lacking a constitutive *R. felis*-infection demonstrated favorable host-pathogen associations for horizontal transmission. The initial report showed that uninfected cat fleas were able to acquire *R. felis* by feeding on a simulated infectious bloodmeal, and this newly acquired infection persists the remainder of the vectors’ lifespan [16]. Following *R. felis* acquisition in previously uninfected cat fleas, the infection then disseminates from the gut to the hemocoel and other tissues before reaching the salivary glands [17•]. Subsequent transmission of *R. felis* to vertebrate hosts is based on serum samples positive to rickettsial antigen and to a lesser extent PCR-positive tissue samples, including blood, resulting from exposure to infected cat fleas (reviewed in [10]). Ultimately, horizontal transmission of *R. felis* was demonstrated through a shared bloodmeal between *R. felis*-infected and uninfected cat fleas in an artificial host system [18•]. Contrary to other vector-borne pathogens, there appears to be no correlation between rickettsial distribution in flea tissues and distinct transmission routes, i.e., horizontal transmission events occur well before the spread of *R. felis* to flea salivary glands (authors’ unpublished data).

The majority of our current understanding of the life cycle of *R. felis* in nature is derived from *R. felis*/*C. felis* laboratory models. The dilemma in this transmission cycle is the subsequent acquisition of viable *R. felis* by cat fleas from vertebrate hosts to complete the “flea to mammal to flea” succession comparable to other insect-borne rickettsial pathogens. Transmission of *R. felis* from cat fleas to vertebrate hosts is presumed to occur through infectious flea bite and potentially infected flea feces, which are also comprised of rickettsiae [16]. Among the mammalian species found to be seropositive or PCR-positive for *R. felis* in nature include cats, dogs, opossums, raccoons, rodents, and humans [10, 19–22]. A definitive mammalian host with a systemic *R. felis* infection has not been identified and may vary by geographic location (e.g., lack of marsupials in Africa, Asia, and Europe) and distribution of arthropod vectors (e.g., sites that have few, if any, cat fleas) [10, 23]. A recent study generated *R. felis*-infected BALB/c mice via an artificial route, and subsequently produced infectious *Anopheles gambiae* mosquitoes that caused transient rickettsemia in naïve mice [24]; nevertheless, naturally infected mammalian blood or tissues have never been shown as a source of *R. felis* infection from vertebrate to arthropod hosts.

**Fig. 1:**
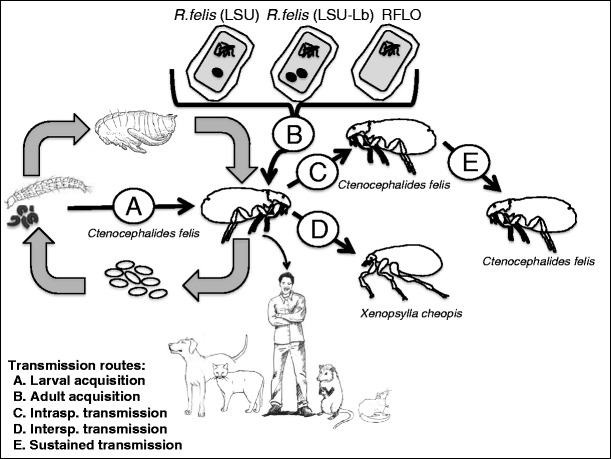
The proposed and described transmission routes necessary for persistence and maintenance of *R. felis* infections within the environment. (*A*) Vertical non-transovarial transmission, i.e., larval acquisition by infectious adult feces, of *R. felis* within cat flea colonies requires experimental confirmation. (*B*) Adult acquisition bioassays with *R. felis* str. LSU and LSU-Lb resulted in infected cat fleas; however, acquisition bioassays with RFLOs have not been attempted. (*C*) Intraspecific transmission of *R. felis* between co-feeding cat fleas was demonstrated both in an artificial system and on a vertebrate host. (*D*) Interspecific transmission of *R. felis* between co-feeding cat fleas and rat fleas was observed on a vertebrate host. (*E*) Sustained transmission of *R. felis* by co-feeding was demonstrated by the continuous spread of infection to newly emerged uninfected cat fleas in an artificial system over the course of 4 weeks

The transmission biology of flea-borne spotted fever is complicated further by the progressive accumulation of field surveys reporting molecular detection of this infectious agent from other vectors, i.e., more than 40 additional species of fleas, ticks, mites, and mosquitoes (Table 1) [4]. Given the infrequency of a systemic vertebrate infection, the presence of *R. felis* in these additional arthropod species is unclear. Successful transmission of pathogens between actively blood-feeding arthropods in the absence of a disseminated vertebrate infection has been demonstrated (reviewed in [25]). This transmission event, referred to as co-feeding, is reliant on the temporal and spatial dynamics of infected and uninfected arthropods as they blood feed. The infected arthropod is both the vector and the reservoir for the pathogen, while the vertebrate acts as a conduit for infection of naïve arthropods. The potential for co-feeding transmission of *R. felis* between cat fleas was demonstrated with the use of a shared bloodmeal in an artificial host system [18•]. Recently, both intra- and interspecific transmission of *R. felis* between co-feeding arthropods on a vertebrate host was demonstrated (Fig. 1C and D) [26•]. Analyses revealed that infected cat fleas transmitted *R. felis* to naïve cat fleas and Oriental rat fleas (*Xenopsylla cheopis*) via flea bite on a non-rickettsemic vertebrate host [26•]. Also, cat fleas infected by co-feeding were infectious to newly emerged uninfected cat fleas in an artificial system (Fig. 1E) [26•]. Furthermore, a stochastic model was utilized to demonstrate that co-feeding is sufficient to explain the enzootic spread of *R. felis* among populations of the biological vector [26•]. These results implicate cat fleas in the spread of *R. felis* among different vectors, and the demonstration of co-feeding transmission of *R. felis* through a vertebrate host represents a novel transmission paradigm for insect-borne *Rickettsia*.

**Table 1:** Geographic distribution of *R. felis* in wild-caught arthropods since 2009 review [10]

Country	Vector	Prevalence of infection	Reference
Albania	*Ctenocephalides felis*	3 % (10/371)	[71]
Algeria	*Archeopsylla erinacei*	96 % (316/331)	[72]
	*Xenopsylla cheopis*, *Leptopsylla segnis*	15 % (10/69)	[73]
Australia	Fleas	ND	[74]
	*C. felis*	ND	[55]
	*Liposcelis bostrychophila*	ND	[75]
Brazil	*C. felis*	38 % (268/701)	[61]
	*Amblyomma humerale*	14 % (1/7)	[76]
	Ticks and fleas	ND	[77]
	*C. felis*	ND	[78]
Chile	*Rhipicephalus sanguineus*	ND	[79]
China	*Eulaelaps stabularis*	ND	[80]
	*C. felis*	95 % (57/60)	[81]
	*R. sanguineus*	10 % (15/146)	
	*Linognathus setosus*	16 % (6/37)	
	*Anopheles sinensis*, *Culex pipiens*	6 % (25/428)	
Colombia	*C. felis*, *Ctenocephalides canis*, *Pulex irritans*	ND	[82]
Costa Rica	*C. felis*	ND	[83]
	*C. felis*	ND	[84]
Côte d’Ivoire	*Anopheles gambiae*	1 % (1/77)	[85]
Cyprus	*X. cheopis*	1 % (4/400)	[86]
Czech Republic	Fleas	18 % (6/33)	[87]
Democratic Republic of Congo (Kinshasa)	*C. felis*	95 % (37/39)	[88]
	*C. canis*	42 % (10/24)	
	*C. felis*	57 % (13/23)	[89]
Democratic Republic of Congo (Ituri)	*C. felis*	23 % (15/64)	[89]
	*Leptopsylla aethiopica*	9 % (1/11)	
	*Echidnophaga gallinacea*	5 % (1/21)	
Ethiopia	Fleas	21 % (63/303)	[90]
	*C. felis*	100 % (3/3)	[91]
	*P. irritans*	43 % (23/53)	
	Fleas	ND	[92]
France	*A. erinacei*	99 % (128/129)	[93]
	*A. erinacei*	11 % (2/19)	[94]
Gabon	*Aedes albopictus*	3 % (3/96)	[95]
Guatemala	*C. felis*	ND	[83]
Hungary	*C. felis*	ND	[96]
Indonesia	*X. cheopis*	ND	[97]
Italy	*C. felis*	26 % (34/132)	[57]
	Fleas	ND	[98]
	*C. felis*	12 % (38/320)	[99]
	*C. felis*	31 % (9/29)	[100]
Ivory Coast	*C. canis*	50 % (1/2)	[101]
Kenya	*X. cheopis*, *C. felis*, *C. canis*, *P. irritans*, *E. gallinacea*	ND	[49•]
Korea	*Ctenophthalmus congeneroides*, *Stenoponia sidimi*, *Rhadinopsylla insolita*	ND	[102]
Laos	*C. canis*, *C. felis*, *Ctenocephalides orientis*	59 % (13/22)	[103]
Lebanon	*C. felis*	16 % (17/104)	[104]
	*C. felis*	44 % (8/18)	[105]
Malaysia	*C. felis*	32 % (57/177)	[22]
	*C. felis*	4 % (4/95)	[106]
	*C. fels*	75 % (337/450)	[107]
Mexico	*C. felis*	25 % (1/4)	[108]
	*Polygenis odiosus*	33 % (1/3)	
Morocco	Fleas	20 % (112/554)	[109]
New Caledonia	*C. felis*	81 % (17/21)	[110]
Netherlands	*C. canis*, *C. felis*	ND	[111]
Panama	*C. felis*	35 % (7/20)	[112]
Peru	*C. felis*	67 % (2/3)	[113]
Reunion Island	*X. cheopis*, *Xenopsylla brasiliensis*	2 % (5/205)	[114]
Senegal	*Aedes luteocephalus*	<1 % (1/203)	[33]
	*Anopheles arabiensis*	1 % (2/154)	
	*Anopheles ziemanni*	14 % (1/7)	
	*Anopheles pharoensis*	10 % (1/10)	
	*Anopheles funestus*	29 % (2/7)	
	*Mansonia uniformis*	25 % (2/8)	
	*Cimex hemipterus*	3 % (1/39)	
Slovakia	*Ctenophthalmus agyrtes*, *Ctenophthalmus solutus*, *Ctenophthalmus uncinatus*, *Nosopsyllus fasciatus*	11 % (34/315)	[115]
Spain	*C. felis*	26 % (20/118)	[116]
	*C. felis*	44 % (34/78)	[117]
	*C. felis*	3 % (2/76)	[118]
Taiwan	*C. felis*	ND	[119]
	*C. felis*	21 % (90/420)	[120]
	*Stivalius aporus*, *Acropsylla episema*	1 % (2/160)	[121]
Tunisia	*C. felis*	9 % (2/22)	[122]
	*C. felis*	<1 % (1/322)	[123]
Turkey	*Rhipicephalus bursa*	ND	[124]
United Republic of Tanzania	*C. felis*	65 % (13/20)	[89]
	*C. canis*	71 % (5/7)	
	*Ctenophthalmus calceatus*	25 % (5/20)	
USA	*C. felis*	ND	[125]
	*C. felis*, *P. irritans*, *X. cheopis*, *E. gallinacea*, *Diamanus montanus*	ND	[126]
	*Amblyomma maculatum*	ND	[127]
	*X. cheopis*	ND	[31]
	*L. bostrychophila*	ND	[41]
	*Carios capensis*	ND	[128]
	*C. felis*, *P. irritans*, *X. cheopis*, *E. gallinacea*, *Diamanus montanus*, *L. segnis*	ND	[129]
	Fleas	ND	[130]
Uruguay	*C. canis*, *C. felis*	41 % (27/66)	[131]
West Indies	*C. felis*	ND	[132]

*ND* not determined

### Epidemiology of *R. felis*

Flea-borne spotted fever is considered an emergent global threat to human health, with cases likely underestimated due to similarities in clinical signs with other febrile illnesses (e.g., fever, rash, headache, and myalgia) and limited access to appropriate laboratory tests (e.g., molecular diagnostics) [4, 10, 15]. The first human case of *R. felis* infection was misdiagnosed as flea-borne endemic typhus (*Rickettsia typhi*) because the available serological reagents were unable to distinguish between the two rickettsial species [6]. A retrospective investigation for *R. felis* among endemic typhus patients was initiated because field surveys revealed the presence of this agent within suspected vectors and mammalian hosts of *R. typhi* in the USA [27–29]. Comparable to endemic typhus, serological and molecular analyses have implicated cat fleas and Virginia opossums (*Didelphis virginians*) as respective vectors and hosts of *R. felis* in suburban regions of California and Texas [21, 27, 29]. The suburban cycle of endemic typhus is unique to the USA due to urban expansion into suburban environments and most likely supplementary to the classic association of *R. typhi* with rat fleas and commensal rats (*Rattus* sp.) [30]. Interestingly, a recent survey revealed a higher prevalence of *R. felis* among Oriental rat fleas and Norwegian rats (*Rattus norvegicus*) than *R. typhi* in endemic typhus areas of Los Angeles [31]. It is unclear whether this urban focus was newly established or represents an expansion of a persistent low-level exposure rate of rat populations to *R. felis*-infected fleas. The vulnerability of human populations to flea-borne rickettsiae is of particular concern in developed countries where aggressive pest management programs may not control for ectoparasites, which can result in the relocation of arthropods to new hosts (e.g., humans and their pets) following rodent extermination. Given the indiscriminate feeding habits of cat fleas [15], *R. felis* is essentially a household rickettsiosis in human populations where peri-domestic animals (e.g., cats, dogs, opossums) are in close contact.

Much of the latest work concerning the epidemiology of *R. felis* has been conducted almost exclusively in Africa due to the considerable frequency of flea-borne spotted fever in hospitalized febrile patients. In sub-Saharan Africa, *R. felis* is described as a common (3–15 %) cause of illness among patients with “fever of unknown origin” in malaria-endemic regions [20, 32, 33]. Remarkably, the incidence of human *R. felis* infections was higher than that of malaria in two of the studied villages of Senegal [32]. This high proportion of *R. felis* infections reported within the last 5 years is in stark contrast to the total number of infections (∼100 human cases) documented worldwide [4]. Again, although *R. felis* is classified as an emerging pathogen, it is unclear whether this increased incidence in Africa reflects an overall trend or represents an endemic state previously unknown for this disease. Commonalities (e.g., geographic distribution, seasonality, target population, incidence of relapses or re-infections, and asymptomatic infections) were observed between the epidemiology of *R. felis* and *Plasmodium falciparum* infections in Africa, which were initially hypothesized to coincide because of a proposed common vector, *Anopheles* mosquitoes [33]. At the time of the Mediannikov et al. [33] publication, the role of *Anopheles* in the transmission of *R. felis* was ambiguous; however, the transmission potential of *R. felis* by *A. gambiae* (the primary malaria vector in sub-Saharan Africa) was recently demonstrated in a simulated model [24]. Other arthropods infected with *R. felis* in Africa include numerous species of fleas, mosquitoes, and mites, as well as an individual bed bug [33]. The vertebrate reservoir host responsible for maintenance of *R. felis* in Africa is unknown, but molecular evidence for the presence of *R. felis* in African apes (chimpanzees, gorillas, and bonobos) was derived from PCR-positive stool samples [34]. It was suggested that similar to malaria and other rickettsial species (e.g., louse-borne epidemic typhus), the reservoir host of *R. felis* in Africa might be primates, including humans [34]. As such, human fecal samples collected from two Senegalese villages with documented *R. felis* infections were PCR-positive for rickettsial DNA [35]. Conversely, it was demonstrated that for predatory apes (chimpanzees and bonobos), the ingestion of an infected prey species and associated ectoparasites might contribute significantly to the presence of parasite nucleic acids in fecal samples and caution should be used when interpreting these molecular analyses [36].

### Genetic Diversity of *R. felis*

Historically, the genus *Rickettsia* (Rickettsiaceae) was designated as typhus group (TG) or spotted fever group (SFG) rickettsiae; however, *R. felis* displayed phenotypic oddities that confounded its categorization as either TG or SFG, e.g., association with an insect, hemolytic activity, actin-based motility, transovarial maintenance in the vector host, and serological cross-reactivity [37]. Additionally, genetic analyses of *R. felis* revealed a large genome size relative to other rickettsiae, and the presence of plasmids [38]. Combined analyses of genome and biological characteristics suggested that additional groups exist within the genus *Rickettsia*, including a sister clade of the SFG now known as the transitional group (TRG) and a non-pathogenic clade, thought to be basal to all other groups, called the ancestral group (AG) [37]. *R. felis* is a member of the TRG rickettsiae, which may explain certain anomalies (e.g., lack of a definitive mammalian host) as this bacterium continues to undergo major life history transitions.

Several strains of *R. felis* have been isolated from colonized and wild-caught arthropods [39, 40], including the non-hematophagous, parthenogenic booklouse *Liposcelis bostrychophila* (Insecta: Psocoptera) [41]. In the booklouse host, *R. felis* is an obligate mutualist required for the early development of the oocyte and is maintained 100 % transovarially [42, 43]. Given that flea-borne strains are considered facultative parasites of the vector, distinct strains of *R. felis* employ different transmission routes for sustained infection within unique arthropod populations [44]. In an effort to determine whether genetic variability determines *R. felis* host specialization, the sequenced genomes of two strains, *R. felis* (str. LSU-Lb) isolated from a booklice colony and *R. felis* (str. LSU) isolated from a cat flea colony, were compared to the flea-derived *R. felis* reference strain (str. URRWXCal2) [44]. Sequence analyses revealed genomic heterogeneity across the three strains of *R. felis*, suggesting that spatial isolation (str. URRWXCal2 vs. str. LSU) and potential host specialization (flea vs. booklouse) have resulted from genetic divergence [44]. Specifically, the discovery of a second, unique plasmid (pLbaR) in the *R. felis* str. LSU-Lb assembly provides evidence for host-specific strain variation [44]. This discovery coincides with other studies that demonstrated differences in plasmid numbers between *R. felis* strains, with some strains having no plasmids and others having two [45, 46]. Towards this understanding, experimental bioassays were generated to determine acquisition of *R. felis* str. LSU-Lb by a colony of cat fleas, as well as subsequent prevalence and infection load dynamics (Fig. 1B). Surprisingly, not only did cat fleas become infected with the booklice strain of *R. felis*, but there were also negligible differences in prevalence and infection loads between both strains within the same cat flea colony. Additionally, similar to *R. felis* str. LSU, no overt fitness effect on cat fleas infected with *R. felis* str. LSU-Lb was observed, including the production and development of F_1_ progeny (authors’ unpublished data). Thus, the selective forces operating on *R. felis* genomes from strains associated with different arthropod vectors remain unknown and require further examination.

Within the last decade, numerous reports have identified *R. felis*-like organisms (RFLOs) in different arthropods, including cat fleas (Table 2), throughout the world based on multilocus sequence typing (MLST). A gene sequenced-based criterion was proposed for the identification of *Rickettsia* isolates at the genus, group, and species level [47]. As such, the number of newly identified *Rickettsia*, specifically RFLOs, has dramatically increased since this recent designation. The proposed genetic guidelines rely on similarities (i.e., percent homology) in the sequences of the 16S rRNA (*rrs*) (≥99.8 %) gene and four protein-coding genes, the *gltA* (≥99.9 %), *ompA* (≥98.8 %), and *ompB* (≥99.2 %) genes and gene D (≥99.3 %) to existing *Rickettsia* species [47]. The concern with this approach is that 0.2 % divergence in the *rrs* gene is the borderline for separation of 2 *Rickettsia* species, whereas 1 % divergence is known to mark the borders of naturally occurring bacterial species [48]. For example, two recently described *Rickettsia* species isolated from cat fleas, *Candidatus* Rickettsia asemboensis and *Candidatus* Rickettsia senegalensis, showed 99.5 and 99.65 % similarity to the *rrs* gene in validated species of *R. felis*, respectively [49•, 50]. Given the potential for genetic diversity of *R. felis* isolates due to spatial isolation, a more suitable approach to justify the separation of RFLOs into species may be to seek ecological, genomic, or phenotypic differences among the major clusters resolved by MLST [48]. Recently, the whole-genome of *Candidatus* Rickettsia asemboensis was sequenced [51], and future comparative analyses may reveal genotypic differences responsible for phenotypic characteristics.

**Table 2:** Geographic distribution of RFLO in wild-caught arthropods

Country	Vector	Prevalence of infection	Reference
Brazil	*Ctenocephalides felis*	ND	[78]
China	*Eulaelaps stabularis*	ND	[80]
Côte d’Ivoire	*Anopheles gambiae*, *Anopheles melas*	7 % (5/77)	[85]
Costa Rica	*C. felis*	ND	[83]
Croatia	*Haemaphysalis sulcata*	23 % (23/101)	[133]
Czech Republic	Fleas	3 % (1/33)	[87]
Ecuador	*C. felis*	100 % (8/8)	[134]
Egypt	*Echidnophaga gallinacea*	100 % (12/12)	[135]
	*Ornithonyssus bacoti*	ND	[136]
France	*Archaeopsylla erinacei*	50 % (2/4)	[105]
Gabon	*Ctencephalides canis*	100 % (12/12)	[105]
	*An. gambiae*	1 % (1/88)	[85]
	*An. melas*	9 % (6/67)	
Germany	*Archaeopsylla erinacei*	96 % (144/150)	[137]
Hungary	*Pulex irritans*	ND	[96]
India	Fleas	78 % (7/9)	[138]
	*C. felis*	73 % (56/77)	[139]
Iran	*Pediobius rotundatus*	20 % (1/5)	[140]
Israel	*Xenopsylla ramesis*, *Synosternus cleopatrae*	ND	[141]
Japan	*C. felis*	39 % (26/67)	[142]
Kenya	*Xenopsylla cheopis*, *C. felis*, *C. canis*, *P. irritans*, *E. gallinacea*	ND	[49•]
	*C. canis*, *C. felis*	ND	[143]
Malaysia	*C. felis*	3 % (6/209)	[144]
Peru	*C. felis*	96 % (71/74)	[145]
Portugal	*Ornithodoros erraticus*	ND	[146]
Senegal	*Synosternus pallidus*	91 % (31/34)	[147]
	*Glossina morsitans*	100 % (78/78)	[148]
	*C. felis*	17 % (5/29)	[50]
Slovakia	*Ctenophthalmus agyrtes*, *Ctenophthalmus solutus*, *Ctenophthalmus uncinatus*, *Nosopsyllus fasciatus*	11 % (34/315)	[115]
Spain	*C. canis*, *C. felis*	28 % (25/88)	[149]
Taiwan	*Leptotrombidium* chigger mites, *Ixodes granulatus*, Mesostigmata mites	ND	[150]
Thailand	*C. canis*, *C. felis*	43 % (66/152)	[151]
Thai-Myanmar border	*C. canis*, *C. felis*	4 % (4/54)	[152]
USA	*C. felis*	100 % (19/19)	[153]
	*C. felis*	ND	[154]
	*Carios capensis*	ND	[128]

*ND* not determined

### Prospective Research for *R. felis*

The transmission routes required for persistence and maintenance of *R. felis* infections in endemic-disease foci remains unclear (Fig. 1A–E). Excretion of viable rickettsiae in the feces of infected arthropods is crucial in transmission cycles for both louse-borne epidemic typhus (*Rickettsia prowazekii*) and flea-borne endemic typhus (*R. typhi*) [30, 52]. The direct inoculation of fecal bacteria by scratching at the bite site constitutes as a persistent source of infection from arthropod to vertebrate hosts. Although *R. felis*-infected cat fleas generate feces with detectable levels of rickettsial transcript [16], the transfer of bacteria from freshly deposited adult feces to susceptible vertebrates has not been demonstrated. Another flea-borne pathogen, *Bartonella henselae*, achieves successful transmission from adult fleas to their progeny via vertical non-transovarial transmission [53]. Vertical transmission of *Bartonella* species was demonstrated, but a previous study showed the absence of transovarial transmission of *B. henselae* within flea colonies [54]; however, when flea larvae were exposed to *Bartonella*-positive adult flea feces, then the larvae acquired an infection that was maintained through adulthood [53]. Thus, vertical non-transovarial transmission of *R. felis* should be tested within cat flea colonies as an additional route of pathogen maintenance in vector populations (Fig. 1A).

The lack of a description of a definitive vertebrate host impedes epidemiological studies of *R. felis* throughout the world. Doubts have been raised about whether *R. felis* transmission from mammal to arthropod occurs given the efficiency of pathogen transfer between co-feeding fleas without a systemic vertebrate infection [26•]; however, field surveys frequently identify mammalian hosts (e.g., cats, dogs, opossums, rodents) as either seropositive or PCR-positive for *R. felis* infections in endemic disease foci. Transmission of *R. felis* within cat flea colonies has proved variable and adaptable, with decreased colony prevalence signaling to increase infection burdens in individual fleas [13]. Thus, only occasional amplification from vertebrate hosts may be needed to enhance or maintain *R. felis* in nature. The latest reports from urban environments have emphasized the potential of domestic cats and dogs as mammalian reservoirs of *R. felis* infections [55–61], while studies from uninhabited localities suggest the importance of rodents and opossums [22, 62]. Accordingly, it appears that a peri-domestic cycle exists for *R. felis* where components of this enzootic cycle are present, e.g., free-ranging cats and dogs, commensal rodents and opossums, and associated flea species. Future studies should address Koch’s postulates to identify *R. felis* as the causative agent of vertebrate infection, specifically isolation of *R. felis* for culture from these proposed reservoir hosts.

Recently, *R. felis* infections in febrile and afebrile patients were diagnosed by PCR detection in human blood samples [33, 63]; thus, it was proposed that perhaps humans could be the natural reservoir for *R. felis*, as they are for another insect-borne rickettsial species (*R. prowazekii*). The transmission cycle for *R. prowazekii* is louse to human to louse, with lice ingesting bacteria by blood-feeding on infected humans and subsequently transferring the bacterium to humans by excretion of infectious feces at the bite site [52]. A delayed complication of *R. prowazekii* is Brill-Zinsser disease, or recrudescent typhus, in which mild symptoms reappear after a latent period [52]. Humans with recrudescent typhus are still capable of infecting lice and spreading the disease [52]. Similarly, *R. felis* DNA was detected in the blood of a patient at multiple time points over a 1.5-month interval. While this initial observation suggests episodic rickettsial infection (relapse or reinfection) in humans, these samples were taken from a child in the absence of antimicrobial therapy [32]. The occurrence of relapses or reinfections of *R. felis* should be investigated further with adult patients administered antibiotic treatment. Additional studies reported that not all patients diagnosed as PCR-positive for *R. felis* infection generated anti-rickettsial antibodies, which researchers proposed supports the notion of a recurrent infection [33, 64]; however, supplementary data may marginalize diagnoses of *R. felis* infection based on PCR-positive blood samples. For example, *R. felis* DNA was detected in skin swabs from healthy individuals in a Senegal village where roughly 7 % of the villagers possess an *R. felis* infection [65, 66]. This study highlights the potential for blood samples from afebrile patients to become polluted by skin surface contaminants prior to molecular analyses [65]. Furthermore, the discovery of *R. felis* in blood samples from asymptomatic persons challenges existing paradigms about pathogenic rickettsiae. Such as, the magnitude of rickettsial growth required for PCR detection in the bloodstream of patients is typically fatal, yet these afebrile individuals had no adverse symptoms [67]. Rickettsioses in febrile and afebrile persons should be confirmed by culture, but as stated previously *R. felis* has not been isolated from a vertebrate host, even in severe human cases. Thus, a human isolate must be obtained before conclusions are drawn on the role of people in *R. felis* epidemiology.

The genetic diversity within the *R. felis* genotype appears to be vast, with different isolates shown to consist of unique individual qualities. Whether RFLOs warrant species designation is unclear, but there are disparities among this genogroup that may lead to a microbial-dependent influence on *R. felis* prevalence. For example, interspecific competition of rickettsiae in ticks is well documented, with a primary infection responsible for the interference or blocking of a secondary infection [68–70]. Thus, the high prevalence of RFLOs in areas where *R. felis* infections appear low or absent may be due to an interference event followed by perpetuation of the primary infection within a closed arthropod population. The transmission biology as well as the pathogenicity of RFLOs is unknown, but these organisms are detected in arthropods known to bite humans. Future work with RFLOs should identify, if any, phenotypic characteristics associated with genotypic diversity and focus on acquisition, dissemination, and transmission of these organisms by their respective arthropod hosts (Fig. 1B).

## Conclusions

Every year, there are new reports of arthropod, animal, and human cases of *R. felis* from additional countries, and the influx of RFLOs may result in a similar trend. Active surveillance of *R. felis* infections among hospitalized febrile patients will determine when an endemic state has been reached by this emerging pathogen, as well as indicate spread to populations outside of endemic disease foci. Advance genetic analyses of *Rickettsia* species should include criteria for ecological, genomic and phenotypic differences in addition to sequence homology. In order to determine the specific roles of both the vertebrate and arthropod host in the transmission cycle of *R. felis*, it is critical to continue the development and implementation of molecular tools and bioassays necessary for more accurate risk assessment and efficacious control measures.
